# Supplementary material for: The Small RNA Universe of Capitella teleta
Source: Front Mol Biosci. 2022 Feb 25;9:802814. doi: 10.3389/fmolb.2022.802814 (PMC8915122; doi:10.3389/fmolb.2022.802814)
Supplement: Supplementary file 1 [file DataSheet1.ZIP › Supplement/known/CAPTEscaffold_273_16225.pdf]

Provisional ID	:	CAPTEscaffold_273_16225
Score total	:	78.8
Score for star read(s)	:	3.9
Score for read counts	:	69.5
Score for mfe	:	0.8
Score for randfold	:	1.6
Score for cons. seed	:	3
Total read count	:	148
Mature read count	:	107
Loop read count	:	0
Star read count	:	41

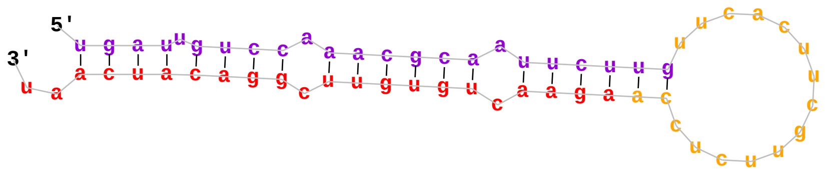

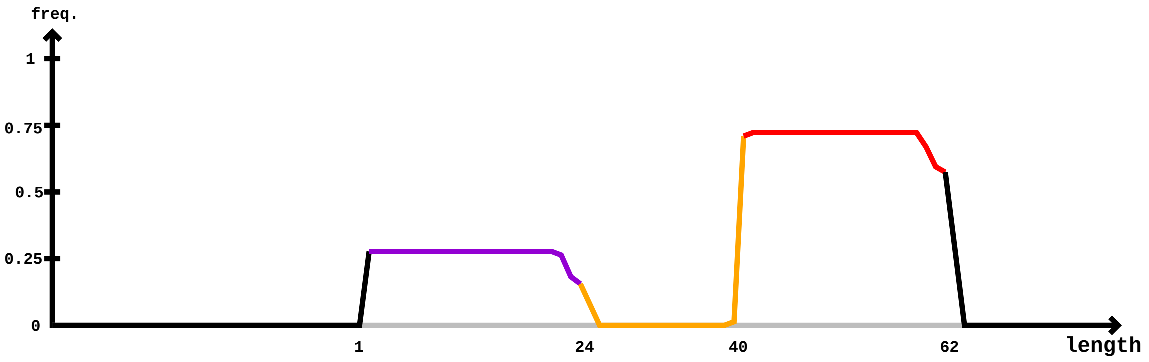


```
                                     Star                                      Mature
5'- auguucaguucagcagacagucuuuuagcauugauuguccaaacgcaauucuuguucacuucguucuccaagaacuguguucggacaucaauccuagcaaggcaguaca   -3'   obs
    auguucaguucagcagacagucuuuuagcauugauuguccaaacgcaauucuuguucacuucguucuccaagaacuguguucggacaucaauccuagcaaggcaguaca         exp
    auguucaguucagcagacagucuuuuagcauugauuguccaaacgcaauucuuguucacuucguucuccaagaacuguguucggacaucaauccuagcaaggcaguaca         known
    .((((......)))).((.(((((((((.((((((.((((.((((((.((((((..............)))))).)))))).)))))))))).)))).))))).))...   reads   mm   sample

    ...............................ugauuguccaaacgcaauuc..........................................................   2       0    seq
    ...............................ugauuguccaaacgcaauucu.........................................................   12      0    seq
    ...............................ugauuguccaaacgcaauucuu........................................................   4       0    seq
    ...............................ugauuguccaaacgcaauucuug.......................................................   23      0    seq
    .....................................................................aagaacuguguucggacaucaau.................   2       0    seq
    ......................................................................agaacuguguucggacauc....................   7       0    seq
    ......................................................................agaacugCguucggacauc....................   1       1    seq
    ......................................................................agaacuguguucggacauca...................   11      0    seq
    ......................................................................agaacuguguucggacaucaa..................   3       0    seq
    ......................................................................agaacugAguucggacaucaau.................   1       1    seq
    ......................................................................agaacuguguuUggacaucaau.................   1       1    seq
    ......................................................................agaacuguguucggacaucaau.................   79      0    seq
    .......................................................................gaacuguguucggacaucaau.................   2       0    seq
```
